# A Case of Opium Habit of Six or Eight Years’ Standing Treated Successfully with the Solid Extract of Coca

**Published:** 1882-01-20

**Authors:** John Q. Winfield

**Affiliations:** Virginia


### A CASE OF OPIUM HABIT OF SIX OR EIGHT YEARS' STANDING TREATED SUCCESSFULLY WITH THE SOLID EXTRACT OF COCA.

BY JOHN Q. WINFIELD, M. D., OF VIRGINIA.

Early in September last, Mrs. —— applied to me for treatment of an opium habit of six or eight years' standing. At the time of application she was taking of laudnum daily the equivalent of about forty grains of opium. She was a blonde, somewhat above medium height, with a full round figure, aged 24. In May last she married an estimable man, who was then totally ignorant of her unfortunate habit.

Up to her twenty-second year, this lady had never menstruated *per vaginam*, but had monthly vicarious bloody discharges from the rectum, attended with much pain. These discharges were usually preceded and followed by painful and exhausting diarrhœa. To relieve her periodical sufferings, she had unadvisedly, I presume, resorted to the use of laudanum, until such use became a fixed habit.

An operation for congenital closure of the external os uteri, by Dr. C. C. Henkle and myself, restored the menstrual function to its proper organs, but did not, of course, relieve the opium habit; hence her return to me as above stated. The attempt extending through twenty days, to cure the case by reliance mainly upon the strength of her own will and the extract of coca in 20-grain doses, four times daily, proved a failure. She was, not (Sept. 28th), with her own consent, placed in close confinement, and only one trusty attendant, besides her husband and physician, allowed to enter the room. No opium prescribed. Ordered 20 grains, four times daily, of the extract of coca.

SEPT. 30.—At bedtime suffering extreme—double vision, want of appetite, nausea, diarrhœa, restlessness, twitching of the muscles, pain in the back and joints, formication, begs piteously for relief. Prescribed $\frac{3}{8}$ of a grain of morphia, concealed in a mixture of wine, bismuth and catechu. She slept well during the night, and was calm and comparatively free from suffering the following morning; coca continued.

OCT. 2.—Night. Suffering, but not so severely as before. Prescribed $\frac{1}{4}$ grain of morphia, concealed as before, and ordered the coca to be continued. Slept well during the night.

OCT. 9.—Night. Suffering somewhat from diarrhœa, menorrhagia, formication and pains in the back and limbs. Prescribed $\frac{1}{8}$ grain morphia. Slept most of the night.

OCT. 15.—Throughout the day calm and free from pain and other disturbances. From this time on to the 18th of October she continued to do well without opium. The coca treatment, however, had been steadily kept up. She was now discharged apparently cured, but advised to continue the coca for a while in diminished daily doses, along with a tonic of quinine, strychnia and iron-

At this writing, November 19th, she is much improved in health and appearance, and does not seem to have the least desire for opium.—[Virginia Medical Monthly, December.

